# Higher prevalence of elevated LDL-C than non-HDL-C and low statin treatment rate in elderly community-dwelling Chinese with high cardiovascular risk

**DOI:** 10.1038/srep34268

**Published:** 2016-09-30

**Authors:** YaShu Kuang, Xiaolin Li, Xiaoli Chen, Huimin Sun, Brian Tomlinson, Paul Chan, Liang Zheng, Jinjiang Pi, Sheng Peng, Hong Wu, Xugang Ding, Dingguang Qian, Yixin Shen, Zuoren Yu, Lieying Fan, Ming Chen, Huimin Fan, Zhongmin Liu, Yuzhen Zhang

**Affiliations:** 1 Research Center for Translational Medicine, Key Laboratory of Arrhythmias, Ministry of Education, Shanghai East Hospital, Tongji University School of Medicine, Shanghai 200120, China; 2 Department of Medicine and Therapeutics, The Chinese University of Hong Kong, Hong Kong SAR, China; 3 Division of Cardiology, Department of Internal Medicine, Wan Fang Hospital, Taipei Medical University, Taipei, Taiwan; 4 Gaohang Community Medical Center, 180 Gingao Road, Pudong New area, Shanghai, 201208, China

## Abstract

Lipid levels are increasing in all age groups in the Chinese population, but the use of statin treatment in the elderly is not well documented. We examined serum lipids, statin usage and achievement of lipid goals in 3950 subjects aged ≥65 years. Established CVD was present in 7.77% of participants and increased CVD risk was common. Elevated LDL-C according to CVD risk level was present in 46.70% of all subjects and was more frequent (p < 0.01) than elevated non-HDL-C at 32.58%. With increasing age, LDL-C was unchanged but triglycerides and non-HDL-C decreased and HDL-C increased. Individuals at moderate risk for CVD had higher TC, LDL-C, and non-HDL-C than low-risk subjects, but the values were lower in high- and very-high-risk individuals, probably because of the use of statin which was 28.57% in high-risk subjects with established CVD and 37.60% in very-high-risk individuals, but only 2.62% in those with estimated high-risk and 3.75% in those with high-risk from diabetes. More subjects in each risk group reached the non-HDL-C goal than the LDL-C goal because of the relatively low triglycerides and VLDL-C levels. These findings demonstrate a high prevalence of elevated LDL-C but low rate of statin treatment in elderly community-dwelling Chinese.

China has experienced a rapid economic growth and aging of its population and the resulting increased prevalence of cardiovascular risk factors including tobacco use, unhealthy diet, reduced physical activity, increased serum cholesterol and glucose, obesity, overweight and hypertension has led to a significant increase in the occurrence of cardiovascular disease (CVD) which accounted for more than 37.8% of all deaths in 2010^1^,^2^,^3^. Cholesterol levels have declined in many economically developed countries between 1980 and 2008 and this led to a reduction of coronary heart disease mortality^4^,^5^, although this risk factor is now being replaced by the increase in prevalence of obesity and diabetes. However, cholesterol levels have increased in low and middle income countries including China^6^,^7^. The increase in serum cholesterol was considered to be the major contributor for the rise of coronary heart disease (CHD) mortality in Beijing from 1984–1999 and was responsible for 77% of the increased mortality^8^, and high cholesterol not only increased the risk for CHD but also the risk for ischaemic stroke^9^.

Both low-density lipoprotein cholesterol (LDL-C) and non-high-density lipoprotein cholesterol (non-HDL-C) are recommended as targets for lipid lowering treatment for primary and secondary prevention of atherosclerotic CVD^10^,^11^,^12^,^13^. The role of high-density lipoprotein cholesterol (HDL-C) as a factor protecting against CVD has become controversial in recent years, but one study found that an increase in the large HDL sub-fraction but not the total HDL-C was closely linked with fewer cardiovascular events in patients with stable CHD^14^. Recent guidelines for lipid management recommend statin treatment for people at increased risk of CVD and indicate LDL-C levels as the primary target and non-HDL-C levels as a secondary target with cut off values according to the level of CVD risk^3^,^12^. Studies which examined the use of statins in patients at increased risk of CVD and the attainment of LDL-C or non-HDL-C goals have generally found a substantial proportion do not achieve the goals, but the use of statins in elderly people over age 75 or 80 years is controversial because of lack of evidence from clinical trials in this age group and observational studies showing an association of low cholesterol levels with increased mortality in the elderly.

The prevalence of CVD rises with advancing age and 80% of CVD deaths occur among elderly individuals above age 65 years. Therefore improving cardiovascular prevention will be important to reduce premature death and CVD events and preserve quality of life in this age group. However, many studies of CVD prevention only focused on young and middle-aged populations and very few have targeted elderly populations, especially the very elderly aged over 80 years.

Shanghai is one of seven cities demonstrating aging and tremendous growth of CVD risk factors and their effects on cardiovascular health^15^, thus the SHanghai Elderly Cardiovascular Health Study (SHECHS) was designed to recruit elderly residents aged ≥65 years living in the Pudong Gaohang Community Medical Center region of Shanghai to provide current and reliable data evaluating the CVD risk factor of high cholesterol in an elderly community population which will help to plan for better interventions to reduce the increasing number of CVD events. This article describes the prevalence of CVD and its major risk factor, serum cholesterol and provides details of the treatment and the attainment of LDL-C or non-HDL-C goals according to CVD risk level from low to very high CVD risk groups in the elderly population of a Chinese community. The data from this study will provide information that can help us recognize appropriate health care and risk management strategies for the elderly subjects who are at high risk for CVD. Our follow-up study will combine medical school affiliated hospital with community medical center interventions to establish a better system for management of high-risk elderly people to improve cardiovascular health care and reduce the incidence of CVD.

## Methods

### Study Population

The SHECHS is a community population-based, longitudinal study of non-institutionalized adults aged ≥65 years as described elsewhere^16^. All people aged ≥65 years who were permanent residents in the community were invited to participate in the study by local community leaders and poster advertisements.

The institutional review board of Tongji Medical School affiliated Shanghai East Hospital approved the study protocol, all studies were performed in accordance with relevant guidelines and regulations, and written informed consent was obtained from each participant before data collection.

### Data Collection

The participants attended Gaohang community medical center in the morning after overnight fasting for at least 10 hours and the blood samples were obtained. Samples for serum lipids, glucose, renal and hepatic function, were drawn in tubes without anticoagulant and samples for HbA1c and other tests in tubes containing EDTA. The samples were measured within 2 hours in the Blood Laboratory of Tongji Medical School affiliated Shanghai East Hospital. Serum total cholesterol (TC), LDL-C, HDL-C, fasting serum glucose (FG), and triglycerides (TG) were measured enzymatically on the Roche Cobas8000 c702 Biochemistry system. Blood HbA1c was measured by ion-exchange high-performance liquid chromatography on the ToSoH G8 analyzer.

After blood sampling, the participants were interviewed by trained family doctors of Gaohang community medical center with standard questionnaires to obtain information on demographic characteristics, personal and medical history, and lifestyle risk factors. ECG was recorded and echocardiography was performed and measured by trained specialists of Shanghai East Hospital using a colour Doppler ultrasonic system equipped with a 1.0–5.0 MHz transducer (GE Vivid 7; General Electric Company, New York, USA). Blood pressure and anthropometric measurements including body weight, height, and waist circumference were obtained according to a standardized protocol^17^.

### Study-outcome Definitions

Established CVD was defined as having a history of myocardial infarction (MI), coronary or other arterial revascularization, or stroke and was confirmed by review of the outpatient medical records of primary care in the community health centers^10^. Definite hypertension was defined as an average of two measurements of systolic blood pressure (SBP) ≥140 mmHg or diastolic blood pressure (DBP) ≥90 mmHg, or normal blood pressure with concomitant use of antihypertensive medications^18^,^19^. Definite diabetes was defined as FG ≥7.0 mmol/l or normal FG with concomitant use of insulin or oral hypoglycaemic agents^20^.

### 10-year Estimated Risk of Ischaemic Cardiovascular Diseases

We followed the Chinese Lipid Guideline^21^ and used an equation validated by the USA-PRC Collaborative Study and the China Multicenter Collaborative Study of Cardiovascular Epidemiology (China MUCA) Research Group, which used traditional covariates age, SBP, BMI, TC, diabetes and smoking. As this study assessed an elderly population, we modified the age score with 1 additional score per 10 years after age 70 years and older instead of 1 score per 5 years^9^,^22^ to facilitate looking at the cholesterol control details in different CVD risk groups of this elderly population. The participants were stratified into low (<10%), moderate (10–20%), high (≥20%, or diabetes, or established CVD) and very high risk (established CVD with diabetes) groups according to the validated Chinese 10-year estimated risk of ischaemic CVD^9^.

### Statistical Analysis

Descriptive statistics were calculated for all variables including mean, 95% confidence interval (CI) and percentile values. The prevalence estimates of TC, LDL-C, and non-HDL-C categories were calculated separately for men and women. The treatment and control rates of high lipids were stratified by age and gender. For the continuous variables, t-test was employed to analyze the difference between two groups. If variances were not equal between two groups, a nonparametric test was used instead of the t-test. For the categorical variables, Chi-squared test (χ2-test) was conducted to show the ratio difference between two groups. Differences among multiple group variables were determined by ANOVA and two group variables by LSD-t test. All statistical analyses were performed using SPSS17.0 software (SPSS Inc., Chicago, IL, USA), a two-tailed P value less than 0.05 was considered to be statistically significant.

## Results

### Demographic and Clinical Characteristics of SHECHS Participants

The baseline demographic and clinical characteristics of the SHECHS participants are shown in Table 1. A total of 3950 participants, 1745 male and 2205 female, aged ≥65 years completed the SHECHS baseline examination and there was no significant age difference between men and women. Only 1.00% of women but 30.58% of men were current cigarette smokers. The prevalence of CVD and risk factors was high in the SHECHS participants: 7.77% of participants had established CVD, 75.08% had definite hypertension and 20.73% had definite diabetes. The prevalence of CVD was not significantly different between men and women, but a significant increase in prevalence with increasing age was found with 5.18%, 9.18% and 12.15% having CVD in the 65–69, 70–79 and ≥80 year age groups, respectively (Table 2, P < 0.01).

### Mean Levels of Lipids and Other Cardiovascular Risk Factors by gender and age

The mean levels of TC, LDL-C, HDL-C, TG and non-HDL-C of SHECHS participants were 4.72, 3.12, 1.38, 1.53 and 3.33 mmol/l in men and 5.20, 3.45, 1.52, 1.71 and 3.67 mmol/l in women, respectively, all being higher in women than men (Table 1, p < 0.01). With increasing age, there were significant decreases in triglyceride levels in both sexes and increases in HDL-C in men, but no significant changes in TC, LDL-C (Table 2 and Supplementary Table 1). Non-HDL-C decreased significantly with age in the data for men and women combined (Table 2, p = 0.016).

As for the other cardiovascular risk factors, we found significant increases in SBP but DBP decreased with increasing age in both genders (Table 2 and Supplementary Table 1, P < 0.01), and there was no difference in SBP between men and women but DBP was significantly lower in women (Table 1, P < 0.01). The mean level of FG was 5.68 mmol/l in men and was higher at 5.82 mmol/l in women (Table 1, P = 0.011) and there was a non-significant trend for this to increase with increasing age (Table 2, p = 0.159). Mean HbA1c was higher in women than in men and this increased with increasing age in men (Supplementary Table 1) and the increase was almost significant in the data for men and women combined (Table 2, P = 0.051).

### Elevated lipids and other CVD risk factors according to CVD risk level

The SHECHS elderly participants had a high prevalence of increased CVD risk using the validated Chinese 10-year estimated risk of ischaemic CVD^9^. Moderate CVD risk was present in 21.92% of participants, 28.63% were at high risk and 3.16% at very high risk (Table 3). There was no significant difference in risk levels between genders (Supplementary Table 2). Compared to low CVD risk subjects, the moderate risk individuals had significantly increased mean TC, LDL-C, and non-HDL, but these values were lower in the high risk group and even lower in the very high risk individuals, while HDL-C decreased progressively from low to very high risk individuals (Table 3). The same trend was observed for both men and women (Supplementary Table 2). These figures are consistent with increased frequency of statin use in higher risk subjects that would reduce TC, LDL-C, and non-HDL levels but may not increase HDL-C levels greatly.

The presence of other CVD risk factors also varied according to the CVD risk level as some of the risk factors are used in the CVD risk level calculation. Compared to low CVD risk subjects, the moderate, high and very high risk individuals had significantly increased mean TG, but no difference was found among moderate, high and very high risk group (Table 3). Levels of SBP and DBP showed a similar increased pattern to LDL-C but the highest levels in the high risk group (Table 3). However, both FG and HbA1c increased progressively from low to very high risk subjects (Table 3).

**Figure 1:**
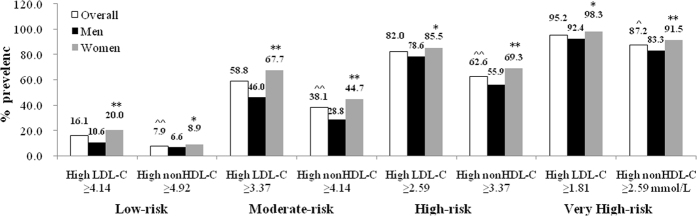
Prevalence of high LDL-C, non-HDL-C in different cardiovascular risk group of Chinese Subjects Aged 65 years and older. LDL-C, low-density lipoprotein cholesterol; Non-HDL-C, sum of LDL-C and VLDL-C calculated as total-C minus HDL-C. *Statistically significantly different from men, p < 0.05. **Statistically significantly different from men, p < 0.01. ^Statistically significantly different from overall LDL-C, p < 0.05. ^^Statistically significantly different from overall LDL-C, p < 0.01.

As the target levels for LDL-C and non-HDL-C differ according to the level of cardiovascular risk as defined by the CVD treatment guidelines^3^,^10^,^12^,^23^, we calculated the prevalence of high LDL-C and high non-HDL-C for each cardiovascular group. The LDL-C goals for low, moderate, high or very high risk are 4.14 mmol/l (160 mg/dl), 3.37 mmol/l (130 mg/dl), 2.59 mmol/l (100 mg/dl) and 1.81 mmol/l (70 mg/dl), respectively, and the goals for non-HDL-C are 4.92 mmol/l (190 mg/dl), 4.14 mmol/l (160 mg/dl), 3.37 mmol/l (130 mg/dl) and 2.59 mmol/l (100 mg/dl), respectively. We found that LDL-C was elevated in 16.08%, 58.83%, 82.03% and 95.20% of the low, moderate, high and very high cardiovascular risk groups, respectively, and elevated overall in 46.70%. These rates of high LDL-C levels were significantly greater than those for the equivalent levels of high non-HDL-C which were 7.93%, 38.13%, 62.63% and 87.20% for low to very high risk groups, respectively, and 32.58% overall (Table 3). These proportions were significantly greater in women than in men (Fig. 1), being 20.00% of women vs. 10.61% of men for high LDL-C in the low risk group (p < 0.01), 67.74% of women vs. 46.02% of men for high LDL-C in the moderate risk group (p < 0.01), 85.45% of women vs. 78.58% of men for high LDL-C in the high risk group (p < 0.05) and 98.30% of women vs. 92.42% of men for high LDL-C in the very high risk group (p < 0.05). A similar trend was found for the prevalence of increased non-HDL-C and the prevalence level was significantly lower than that for increased LDL-C (Fig. 1, P < 0.01, except P < 0.05 in the low risk group).

### Lipid treatment and goal attainment

**Figure 2:**
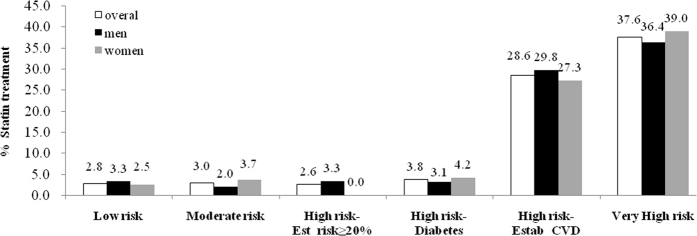
Percentage of statin treatment in different cardiovascular risk group of Chinese Subjects Aged 65 years and older. Est risk = estimated risk, Estab CVD = established CVD.

We further analyzed the lipid treatment and goal attainment according to the Chinese guidelines of prevention and treatment of dyslipidaemia (Supplementary Table 3)^3^. Without statin treatment, the proportion of subjects with LDL-C not at goal was 13.62%, 57.02%, 80.97% and 92.30%, respectively, from low to very high risk individuals, with similar proportions not reaching TC goals but the numbers not reaching non-HDL-C, were lower at 5.23%, 33.33%, 58.79% and 79.48%, respectively (Table 4). The percentage of subjects having statin treatment for low, moderate, high and very high risk individuals was 2.84%, 3.00%, 7.51% and 37.60%, respectively (Table 4 and Fig. 2). The proportion of subjects not reaching LDL-C goals was significantly lower in those receiving statin treatment compared to those not on treatment at 53.45% vs. 30.76%, 58.82% vs. 80.97% and 76.59% vs. 92.30% in the moderate, high and very high risk groups, respectively, and the differences in the proportions of subjects in the low risk groups not reaching LDL-C goals with and without statin treatment were not significant because of small number not at high cholesterol goal, and the proportions of subjects not reaching TC and non-HDL-C goals in the very high risk group were similar in those with and without statin treatment (Table 4). Similar findings were seen in the data from men and women analyzed separately (Supplementary Table 4).

The high risk group includes those with established CVD or equivalent CVD risk of diabetes or 10 year estimated CVD risk >20%, and we found that 27.20% of those with established CVD were on statins, but only 3.75% of those with diabetes, the CVD-equivalent risk, and only 2.62% of those with 10 year estimated CVD risk >20% were on statin (Table 5 and Fig. 2). Without statin treatment, the proportions of high risk subjects not at TC or LDL-C goals were about or over 80%, but with statin treatment the proportion not reaching the LDL-C goal was reduced to 53.84% in those with established CVD and 66.66% in the group with diabetes and 10-year estimated CVD risk >20% (Table 5).

Taken together, these results show that subjects with moderate to very high CVD risk had high levels of LDL-C and low rates of statin treatment, especially in the moderate CVD risk group and the high CVD risk group with 10 year estimated CVD risk >20% and those with diabetes. Even though the rates of statin treatment were 28.57% and 37.60% for those with the high risk of established CVD and very high risk individuals, respectively, a high proportion of these subjects did not achieve the LDL-C target level.

Analysis of the types of statin used in the elderly population showed the most frequently used statin was atorvastatin at 60.95% followed by simvastatin at 21.9%, and of the total participants using atorvastatin 67.97% were using 10 mg and 19.53% were using 20 mg, and 71.73% of the participants were using 20 mg simvastatin (Supplementary Table 5). The individuals not reaching the goal LDL-C of 2.59 mmol/L in the high risk group were 53.84% in those with established CVD and 66.66% in those with diabetes (Table 5). Considering that low doses of statin were mostly used this suggested these small statin doses may be relatively effective in controlling high cholesterol in these elderly subjects.

## Discussion

There was high prevalence of CVD and risk factors among the elderly SHECHS participants. Hyperlipidaemia is the most common modifiable risk factor for CVD. Previously the mortality from CHD in China was only one-tenth of that in western developed countries due to low levels of cholesterol^8^,^24^, but over the last 3 decades the age-standardized mean TC in China increased from 4.2 to 4.5 mmol/l in men and from 4.3 to 4.6 mmol/l in women and has become the major risk factor for CVD^6^,^7^,^8^,^25^,^26^. However, few studies have been conducted in the elderly, especially the very elderly of the Chinese community, and studies on elderly subjects usually had a small sample size.

A cross-sectional study of elderly individuals from a rural community showed highly prevalent and poorly controlled levels of high cholesterol but this included only 1538 participants and few very elderly individuals^12^. The report on a total of 19981 residents aged ≥60 years from the 2010 Chinese Surveillance of Chronic Non-communicable Disease and Risk Factor provided only the prevalence of high cholesterol without detailed lipid lowering treatment and control information^27^. One recent large scale cross-sectional study of a community-based population from China Kadoorie Biobank (CKB) included 91771 adults aged between 60–69 years and 33011 adults aged between 70–79 years which provided the CVD incidence and lipid lowering medication information, but lacked detailed information on lipid fractions^28^.

The SHECHS study recruited 3950 participants aged ≥65 years including 608 very elderly participants aged ≥80 years and found that 7.77% of the participants had established CVD. The mean concentration of TC was 4.72 mmol/l in men and 5.20 mmol/l in women (Table 1), which was a little lower than the TC levels of the 2007 China national survey of 4.85 mmol/l for men and 5.23 mmol/l for women^7^, but close to 5.07 mmol/l (196 mg/dl), the level of the US population in 2010 and 5.14 mmol/l, the recent mean level of the population in Iceland^4^,^29^. The prevalence of TC ≥5.18 mmol/l (200 mg/dl) was 44.8%, suggesting that many Chinese elderly individuals had high serum TC.

The previous prospective Cohort studies in China^3^,^21^ found that TC levels were positively related to CVD events, and the incidence rate of ischaemic stroke in China is two times higher than that of CHD events. The China MUCA research group proposed the concept of ischaemic cardiovascular diseases including ischaemic stroke and validated an equation more accurately reflecting the harmful role of dyslipidaemia on Chinese people’s health. We used this validated equation to observe the lipid control status according to different CVD risk levels and found the prevalence of LDL-C levels above the target for the risk group increased from low to very high-risk groups, but was lower in those on statin treatment, particularly in the high and very high risk groups with established CVD (Tables 3, 4 and 5). Lipid lowering medications were used mainly in those with established CVD, but rarely for those at moderate risk or high CVD risk with a high estimated CVD risk score or having diabetes. This may be because the participants are not aware of their high CVD risk level, the doctors looking after these subjects in the community may not calculate the CVD risk level, or that they have diabetes and the doctors may not be aware that diabetes should be regarded as a CHD risk equivalent. A previous large prospective study of pravastatin in the elderly at risk, PROSPER, found that statin could reduce cardiovascular events and all-cause mortality in the elderly subjects^30^. There were studies reported that lipid lowering medication in China was only used for the treatment of elevated cholesterol in patients with established CVD and the proportion on treatment for high cholesterol was low in those at high CVD risk without established CVD^28^,^29^,^31^. The present study demonstrated that SHECHS elderly participants had a high incidence of CVD, but the percentage on statin treatment was 5.31% overall and for low-, moderate- and high-risk individuals 2.84%, 3.00%, 7.51% respectively, whereas for high-risk subjects with established CVD it was 28.57% and very-high-risk individuals 37.60% (Table 4, 5 and Fig. 2), which is greater than the 1.4% statin use in patients with CVD reported in the Chinese Kadoorie Biobank (CKB) study^28^, but less than the 12.0% statin treatment in the 1999–2000 year US population survey^32^.

Based on the recent Heart Outcomes Prevention Evaluation (HOPE)–3 trial^33^, many of the subjects in the moderate risk for CVD group would also benefit from statin therapy so there appears to be a considerable need to increase statin usage in this elderly community population.

The TC level includes the total blood lipoprotein cholesterol and is often used in screening programs and risk assessment algorithms. LDL-C accounts for more than 75% of atherogenic lipoproteins, therefore most dyslipidaemia guidelines identify LDL-C as the main factor for CVD risk evaluation and the primary target of therapy^10^,^11^,^12^,^13^. The recent National Lipid Association (NLA) guideline^13^ concluded that non-HDL-C levels were more strongly associated with future risk of CVD events than LDL-C and recommended the use of non-HDL-C levels as targets of therapy as well as LDL-C. Our study found that in the elderly participants TG levels were relatively low and declined significantly with increasing age in both genders (Table 2) and this would also apply to very low-density lipoprotein cholesterol (VLDL-C) levels which are calculated from the TG levels. Consequently, the prevalence of non-HDL-C ≥4.92 mmol/L was significantly less than that of the equivalent LDL-C level of ≥4.14 mmol/L or TC ≥5.18 mmol/L in the low CVD risk group (Fig. 1). Further analysis showed that the proportion not reaching the LDL-C goal was significantly higher than those not reaching the equivalent non-HDL-C goal in all groups from low to very high risk (Table 4). Therefore using criteria such as those in the NLA guideline to define non-HDL-C goals, LDL-C goals would be a more difficult target to reach than non-HDL-C goals in the elderly population of China. Alternatively, the target values for non-HDL-C should be adjusted to account for the lower levels of TG in this population so that non-HDL-C levels could be useful as a secondary goal for therapy in some individuals.

SHECHS participants had a low rate of treatment for high cholesterol, but the proportion of subjects not reaching LDL-C goals was significantly lower in those receiving statin treatment compared to those not on treatment in the high and very high risk groups (Tables 4 and 5). Analyzing the types of statin used showed that 60.95% of patients received atorvastatin, 21.90% received simvastatin and mostly low doses were used in this elderly community population (Supplementary Table 5). A study in a rural community elderly Chinese population^31^ reported that 41.1% of participants with CVD were on lipid lowering treatment and 31.7% achieved good control. This suggested that low doses of statins maybe effective in controlling the high cholesterol in many of these community dwelling elderly individuals. A recent study from the USA also found that in elderly patients with CHD discharged from hospital, the use of statin therapy at discharge was associated with improved long-term outcomes, but high-intensity statin therapy was not associated with incremental benefit over moderate intensity statin therapy in CHD patients >75 years old^34^.

The major limitation of this study is that it is a cross sectional observational study in a community-based population for evaluation of CVD and its major lipid risk factors, high TC, LDL-C and non-HDL-C, and thus the results will only reflect the elderly residents living in the city area rather than from rural communities and longitudinal data relating lipid levels and treatments to cardiovascular and other outcomes are not available yet.

In conclusion, the SHECHS elderly participants had a high incidence of CVD but poor control of high cholesterol especially in moderate-risk and high-risk elderly individuals without established CVD. Low doses of statins were mostly used and appeared relatively effective in reaching LDL-C targets although many subjects would need more intensive treatment to reach the goal. LDL-C goals appeared to be more difficult to reach as a target for statin therapy than non-HDL-C goals as the contribution of triglyceride-rich lipoproteins to non-HDL-C levels diminished with age in this elderly community population. Future best practices for screening and calculating the CVD risk score and proper use of lipid lowering medication for primary prevention should be stressed to achieve good control of hypercholesterolaemia to reduce CVD events more effectively, especially in individuals with moderate and high CVD risk without established CVDs.

## Additional Information

**How to cite this article**: Kuang, Y. *et al*. Higher prevalence of elevated LDL-C than non-HDL-C and low statin treatment rate in elderly community-dwelling Chinese with high cardiovascular risk. *Sci. Rep.* **6**, 34268; doi: 10.1038/srep34268 (2016).

## Supplementary Material

Supplementary Information

## Figures and Tables

**Table 1:** Demographic and Clinical Characteristics of Chinese Subjects Aged over 65 Years Stratified by Gender.

	**All (3950)**	**Men (1745)**	**Women (2205)**	**P value**
Gender (%)		44.22	55.81	
Age (years)	71.96 (71.75–72.16)	71.76 (71.46–72.07)	72.11 (71.83–72.39)	0.097
Education, % (n)				<0.01
<6 year	41.74 (1649)	24.34 (425)	55.51 (1224)	
6–12year	48.09 (1900)	58.48 (1021)	39.86 (879)	
>12 year	10.17 (402)	17.18 (300)	4.63 (102)	
Health habits				
Current cigarette user, % (n)	14.07 (556)	30.58 (534)	1.00 (22)	<0.01
Current alcohol user, % (n)	13.71 (542)	29.32 ( 512)	1.36 (30)	<0.01
Definite hypertension, % (n)	75.08 (2966)	74.21 (1295)	75.78 (1671)	0.267
SBP, mmHg	138.8 (138.3–139.3)	138.4 (137.6–139.3)	139.1 (138.4–139.8)	0.237
DBP, mmHg	81.8 (81.5–82.1)	82.5 (82.1–82.9)	81.3 (80.9–81.7)	<0.01
BMI, kg/m^2^	24.62 (24.51–24.72)	24.46 (24.31–24.61)	24.74 (24.59–24.89)	0.011
Definite diabetes, % (n)	20.73 (819)	19.71 (344)	21.54 (475)	0.156
Awareness of Diabetes, % (n)	78.38 (642)	80.52 (277)	76.84 (365)	0.119
Treatment of Diabetes, % (n)	13.77 (544)	13.69 (239)	13.83 (305)	0.926
FG, mmol/l	5.76 (5.70–5.81)	5.68 (5.59–5.76)	5.82 (5.74–5.90)	0.018
HbA1c, %	6.35 (6.32–6.39)	6.30 (6.25–6.35)	6.39 (6.35–6.44)	<0.01
≥6.5, %	25.82 (1020)	24.24 (423)	27.07 (597)	0.044
≥7.0, %	15.87 (627)	14.72 (257)	16.78 (370)	0.087
Established CVD†	7.77 (307)	9.16 (160)	6.66 (147)	0.056
Lipid profile				
Statin treatment, % (n)	5.31 (210)	5.67 (99)	5.03 (111)	0.392
TC, mmol/l	4.98 (4.95–5.01)	4.72 (4.67–4.76)	5.20 (5.16–5.24)	<0.01
LDL-C, mmol/l	3.30 (3.28–3.33)	3.12 (3.08–3.16)	3.45 (3.41–3.49)	<0.01
HDL-C, mmol/l	1.46 (1.45–1.47)	1.38 (1.36–1.40)	1.52 (1.51–1.54)	<0.01
TG, mmol/l	1.63 (1.59–1.66)	1.53 (1.47–1.58)	1.71 (1.66–1.76)	<0.01
Non-HDL-C, mmol/l	3.52 (3.49–3.55)	3.33 (3.29–3.37)	3.67 (3.63–3.71)	<0.01

Values are mean and 95% confidence interval (CI), or percentages % (number).

Percentage of treatment calculated as medication used in hypertension or diabetes.

Established CVD includes history of MI, coronary or other arterial revascularization, stroke; definitive hypertension defined as SBP ≥140 mmHg or DBP ≥90 mmHg, or normal BP with concomitant use of antihypertensive medications; definitive diabetes as FG ≥7.0 mmol/L or normal blood glucose with concomitant use of hypoglycemic medications.

SBP, systolic blood pressure; DBP, diastolic blood pressure; TC, total cholesterol; LDL-C, low density lipoprotein cholesterol; HDL-C, high density lipoprotein cholesterol; Non-HDL-C, sum of LDL-C and VLDL-C calculated as total-Cholesterol minus HDL-Cholesterol; TG, triglyceride; FG, fasting glucose.

To convert from mmol/L to mg/dl, divide by 0.05551 for glucose, by 0.02586 for TC, HDL-C, and LDL-C, and by 0.01129 for triglycerides.

**Table 2:** Plasma lipids and Other Metabolic Variables in Chinese Subjects Aged over 65 Years Stratified by Age.

Age (years)	65–69	70–79	≥80	P value
	1850	1492	608	
Number	X (95% CI)	X (95% CI)	X (95% CI)	
Established CVD, % (n)	5.18 (96)	9.18 (137)	12.15 (74)	<0.01
Lipid profile				
Statin treatment, % (n)	4.97 (92)	5.42 (81)	6.07 (37)	0.553
TC, mmol/l	5.00 (4.96–5.05)	4.97 (4.92–5.02)	4.96 (4.88–5.04)	0.515
LDL-C, mmol/l	3.31 (3.27–3.35)	3.31 (3.26–3.36)	3.27 (3.20–3.34)	0.643
HDL-C, mmol/l	1.44 (1.42–1.46)	1.45 (1.43–1.47)	1.53 (1.49–1.56)	<0.01
TG, mmol/l	1.70 (1.64–1.76)	1.60 (1.55–1.65)	1.46 (1.40–1.52)	<0.01
Non-HDL-C, mmol/l	3.56 (3.51–3.60)	3.51 (3.46–3.56)	3.43 (3.35–3.50)	0.016
FG, mmol/l	5.70 (5.62–5.79)	5.77 (5.68–5.87)	5.87 (5.70–6.04)	0.159
HbA1c, %	6.32 (3.27–6.36)	6.36 (6.30–6.41)	6.44 (6.34–6.54)	0.051
SBP, mmHg	136.4 (135.7–137.2)	140.5 (139.6–141.4)	141.8 (140.5–143.2)	<0.01
DBP, mmHg	82.4 (82.0–82.8)	81.4 (81.0–81.9)	80.92 (80.1–81.6)	<0.01

Values are mean and 95% confidence interval (CI), or percentages % (number).

Established CVD includes history of MI, coronary or other arterial revascularization, stroke, or peripheral arterial disease; TC, total cholesterol; LDL-C, low density lipoprotein cholesterol; HDL-C, high density lipoprotein cholesterol; Non-HDL-C, sum of LDL-C and VLDL-C calculated as total-C minus HDL-C; TG, triglyceride; FG, fasting glucose, SBP, systolic blood pressure; DBP, diastolic blood pressure.

**Table 3:** Serum lipid levels and other cardiovascular risks according to 10-year estimated risk of atherosclerotic ischemic cardiovascular diseases in elderly individuals.

	Low-risk	Moderate-risk	High-risk	Very High-risk	P value
% (n)	46.27 (1828)	21.92 (866)	28.63 (1131)	3.16 (125)	
Lipid profile					
TC, mmol/l	4.87 (4.83–4.92)	5.23 (5.16–5.29)**	5.01 (4.95–5.075)**	4.71 (4.53–4.90)	<0.01
LDL-C, mmol/l	3.22 (3.18–3.25)	3.52 (3.46–3.589)**	3.30 (3.25–3.36)**	3.08 (2.92–3.25)	<0.01
HDL-C, mmol/l	1.50 (1.48–1.52)	1.47 (1.454–1.50)*	1.41 (1.38–1.43)**	1.32 (1.25–1.38)**	<0.01
TG, mmol/l	1.47 (1.43–1.52)	1.72 (1.64–1.79)**	1.80 (1.710–1.88)**	1.74 (1.60–1.88)**	<0.01
Non-HDL-C, mmol/l	3.37 (3.33–3.41)	3.75 (3.68–3.81)**	3.60 (3.54–3.66)**	3.39 (3.21–3.57)	<0.01
**Overall Prevalence**					
High LDL-C, % (n)	46.70 (1845)				
High Non-HDL-C, % (n)	32.58 (1287)				
Age, years	70.23 (69.96–70.50)	73.49 (73.03–73.95)	73.36 (72.97–73.75)	73.85 (72.67–75.03)	<0.01
FG, mmol/l	4.95 (4.93–4.98)	5.31 (5.27–5.36)**	7.20 (7.04–7.35)**	7.52 (7.12–7.92)**	<0.01
HbA1c, %	5.85 (5.84–5.87)	6.37 (6.32–6.43)**	7.02 (6.93–7.11)**	7.46 (7.22–7.69)**	<0.01
SBP, mmHg	131.5 (130.9–132.2)	144.3 (143.3–145.3)**	146.0 (144.9–147.1)**	141.6 (138.5–144.7)**	<0.01
DBP, mmHg	80.0 (79.6–80.4)	83.3 (82.7–83.8)	83.5 (82.9–84.1)	82.6 (80.9–84.3)	<0.01

Values are mean and 95% confidence interval (CI), or percentages % (number).

10-year estimated risk groups were for low (<10%), moderate, (10–20%), high (≥20%, or established CVD or diabetes) and very high (established CVD plus diabetes) risk group.

SBP, systolic blood pressure; TC, total cholesterol; LDL-C, low-density lipoprotein cholesterol; HDL-C, high-density lipoprotein cholesterol; Non-HDL-C, sum of LDL-C and VLDL-C calculated as total-C minus HDL-C; TG, triglyceride; FG, fasting glucose, BMI, body mass index.

^*^P < 0.05 compared to low risk subject; ^**^P < 0.01 compared to low risk subject.

**Table 4:** Statin treatment and control of dyslipidemia in low to very high CVD risk of Chinese subject aged over 65 years.

	Low risk (n = 1828)		Moderate risk (n = 866)		High risk (n = 1131)		Very High risk (n = 125)	
	No statin	Statin	No statin	Statin	No statin	Statin	No statin	Statin
Statin treatment, % (n)		2.84 (52)		3.00 (26)		7.51 (85)		37.60 (47)
Total TC not at goal	8.09 (148)		52.77 (457)		80.99 (916)		97.60 (122)	
TC not at goal, % (n)	8.22 (146)	3.84 (2)	53.45 (449)	30.76 (8)*	82.12 (859)	67.05 (57)**	97.43 (76)	97.87 (46)
Total LDL-C not at goal	13.45 (246)		56.23 (487)		79.31 (897)		86.40 (108)	
LDL-C not at goal, % (n)	13.62 (242)†	7.69 (4)†	57.02 (479)‡	30.76 (8) * ‡	80.97 (847)‡	58.82 (50)** ‡	92.30 (72)‡	76.59 (36)* †
TG>1.7 mmol/L, % (n)	26.01 (462)	34.61 (18)	36.66 (308)	36.84 (9)	39.19 (410)	34.24 (25)	42.30 (33)	46.80 (22)
HDL-C, % (n)	15.26 (279)		15.81 (133)		20.33 (230)		22.40 (28)	
men<1.0, women <1.2mmol/L	15.37 (273)	11.53 (6)	15.83 (133)	15.38 (4)	21.41 (224)	16.66 (6)	20.51 (16)	25.53 (12)
Total Non-HDL-C not at goal	5.19 (95)		32.90 (285)		57.38 (649)		76.00 (95)	
Non-HDL not at goal, % (n)	5.23 (93)	3.84 (2)	33.33 (280)	19.23 (5)	58.79 (615)	40.00 (34)**	79.48 (62)	70.21 (33)

10-year estimated risk groups were for low (<10%), moderate, (10–20%), high (>20%, diabetes or established CVD) and very high risk (established CVD plus diabetes) group.

TC, total cholesterol; LDL-C, low-density lipoprotein cholesterol; TG, triglyceride; HDL-C, high-density lipoprotein cholesterol; Non-HDL-C, sum of LDL-C and VLDL-C calculated as total-C minus HDL-C.

^*^Statistically significantly different from non-statin treatment group, p < 0.05.

^**^p < 0.01.

^†^Statistically significantly different from non-HDL-C, p < 0.05.

^‡^Statistically significantly different from non-HDL-C, p < 0.01.

**Table 5:** Statin treatment and control of dyslipidemia in detailed classification of high risk population.

	High risk (n = 1131)					
	Estimate risk > 20% (229)		Diabetes (720)		Established CVD (182)	
	No statin	Statin	No statin	Statin	No statin	Statin
Statin treatment, % (n)		2.62 (6)		3.75 (27)		28.57% (52)
Total TC not at goal	85.15 (195)		80.69 (581)		75.27 (137)	
TC not at goal, % (n)	86.09 (192)	50.50 (3)	80.95 (561)	74.07 (20)	81.53 (106)	59.61 (31)*
Total LDL-C not at goal	86.46 (198)		77.91 (561)		75.82 (138)	
LDL-C not at goal, % (n)	86.99 (194)†	66.66 (4)* ‡	78.35 (543)†	66.66 (18)* †	84.61 (110)‡	53.84 (28)** ‡
TG >1.7 mmol/L, % (n)	32.87 (80)	33.33 (2)	41.99 (291)	51.85 (14)	30.00 (39)	23.07 (12)
HDL-C, % (n)						
men <1.0, women <1.2 mmol/L	10.76 (24)	33.33 (2)	25.39 (176)	25.92 (7)	18.46 (24)	11.53 (6)
Total Non-HDL-C not at goal	63.31 (144)		58.75 (423)		42.85 (78)	
Non-HDL not at goal, % (n)	64.12 (143)	33.33 (1)	59.30 (411)	44.44 (12)*	46.92 (61)	32.70 (17)*

TC, total cholesterol; LDL-C, low-density lipoprotein cholesterol; TG, triglyceride; HDL-C, high-density lipoprotein cholesterol; Non-HDL-C, sum of LDL-C and VLDL-C calculated as total-C minus HDL-C.

^‡^Statistically significantly different from non-HDL-C, p < 0.01.

^†^Statistically significantly different from non-HDL-C, p < 0.05.

^*^Statistically significantly different from non-statin treatment group, p < 0.05.

^**^Statistically significantly different from non-statin treatment group, p < 0.01.
