# Supplementary material for: Higher prevalence of elevated LDL-C than non-HDL-C and low statin treatment rate in elderly community-dwelling Chinese with high cardiovascular risk
Source: Sci Rep. 2016 Sep 30;6:34268. doi: 10.1038/srep34268 (PMC5043234; doi:10.1038/srep34268)
Supplement: Supplementary Information [file srep34268-s1.pdf]

Higher prevalence of elevated LDL-C than non-HDL-C and low statin treatment rate in elderly community-dwelling Chinese with high cardiovascular risk

YaShu Kuang*[1], Xiaolin Li*[1], Xiaoli Chen*[1], Huimin Sun[1], Brian Tomlinson[2], Paul Chan[3], Liang Zheng[1], Jinjiang Pi[1], Sheng Peng[1], Hong Wu[4], Xugang Ding[4], Dingguang Qian[4], Yixin Shen[1], ZuorenYu[1], Lieying Fan[1], Ming Chen[1], Huimin Fan[1], Zhongmin Liu[§1], Yuzhen Zhang[§1]

1, Research Center for Translational Medicine, Key Laboratory of Arrhythmias, Ministry of Education, Shanghai East Hospital, Tongji University School of Medicine, Shanghai 200120, China

2, Department of Medicine and Therapeutics, The Chinese University of Hong Kong, Hong Kong SAR, China

3, Division of Cardiology, Department of Internal Medicine, Wan Fang Hospital, Taipei Medical University, Taipei, Taiwan

4, Gaohang Community Medical Center, 180 Gingao Road, Pudong New area, Shanghai, 201208, China

Supplement table 1: Plasma lipids and Other Metabolic Variables in Chinese Subjects Aged over 65 Years Stratified by Age and Gender

Age (years)	65-69	70-79	≥80	
	X (95% CI)	X (95% CI)	X (95% CI)	P value
**Men**	831	667	247	
Established CVD, % (n)	6.49 (54)	10.79 (72)	13.76 (34)	<0.01
Lipid profile				
Statin treatment, % (n)	4.69 (39)	6.74 (45)	6.07 (15)	0.223
TC, mmol/l	4.73 (4.67-4.79)	4.71 (4.63-4.78)	4.70 (4.58-4.82)	0.831
LDL-C, mmol/l	3.12 (3.06-3.18)	3.12 (3.05-3.18)	3.09 (2.99-3.20)	0.880
HDL-C, mmol/l	1.35 (1.32-1.37)	1.39 (1.36-1.42)	1.47 (1.42-1.52)*	<0.01
TG, mmol/l	1.64 (1.56-1.73)	1.45 (1.38-1.51)	1.33 (1.23-1.43)*	<0.01
Non-HDL-C, mmol/l	3.38 (3.32-3.44)	3.31 (3.24-3.38)	3.23 (3.11-3.34)	0.050
FG, mmol/l	5.65 (5.53-5.77)	5.67 (5.54-5.80)	5.78 (5.48-6.07)	0.634
HbA1c, %	6.27 (6.20-6.34)	6.27 (6.19-6.35)	6.47 (6.29-6.64)	0.031
SBP, mmHg	135.9 (134.7-137.07)	140.1 (138.8-141.4)	142.5 (140.2-144.7)	<0.01
DBP, mmHg	83.3 (82.7-84.0)	81.9 (81.3-82.6)	81.1 (79.9-82.3)	0.01
**Women**	1019	825	361	
Established CVD, % (n)	4.12 (42)	7.88 (65)	11.04 (40)	<0.01
Lipid				
Statin treatment, % (n)	5.2 (53)	4.3 (36)	6.0 (22)	0.431
TC, mmol/l	5.22 (5.17-5.28)‡	5.19 (5.12-5.25)‡	5.14 (5.03-5.24)‡	0.310
LDL-C, mmol/l	3.46 (3.41-3.51)‡	3.46 (3.40-3.53)‡	3.40 (3.31-3.49)‡	0.455
HDL-C, mmol/l	1.52 (1.50-1.55)‡	1.51 (1.48-1.53)‡	1.57(1.52-1.61)†	0.057
TG, mmol/l	1.75 (1.66-1.84)	1.73 (1.65-1.80)‡	1.55 (1.47-1.63)†*	0.025
Non-HDL-C, mmol/l	3.70 (3.64-3.76)	3.67 (3.61-3.74)	3.56 (3.46-3.66)	0.076
FG, mmol/l	5.75 (5.64-5.86)	5.86 (5.72-5.99)	5.93 (5.73-6.14)	0.220
HbA1c, %	6.36 (6.29-6.42)	6.43 (6.35-6.51)	6.42 (6.31-6.54)	0.338
SBP, mmHg	136.9 (135.9-137.9)	140.8 (139.6-142.1)	141.4 (139.7-143.0)	<0.01
DBP, mmHg	81.7 (81.2-82.2)†	81.0 (80.4-81.6)	80.7 (79.8-81.6)	0.046

Values are mean and 95% confidence interval (CI), or percentages % (number).

Established CVD includes history of MI, coronary or other arterial revascularization, stroke, or peripheral arterial disease; TC, total cholesterol; LDL-C, low density lipoprotein cholesterol; HDL-C, high density lipoprotein cholesterol; Non-HDL-C, sum of LDL-C and VLDL-C calculated as total-C minus HDL-C; TG, triglyceride; FG, fasting glucose, BMI, body mass index; SBP, systolic blood pressure; DBP, diastolic blood pressure;

* Statistical comparison for linear change across age groups, P<0.05; ** P<0.01

†Statistically significantly different from men, p<0.05; ‡Statistically significantly different from men, p<0.01.

Supplement table 2: Serum lipid levels and other cardiovascular risks according to 10-year estimated risk of atherosclerotic ischemic cardiovascular diseases in elderly individuals

	Low-risk	Moderate-risk	High-risk	Very High-risk	P value
Men, n					
% (n)	43.72 (763)	20.12 (351)	32.38 (565)	3.78 (66)	
Lipid Profile					
TC, mmol/l	4.58 (4.52-4.64)	4.91 (4.81-5.00)	4.82 (4.74-4.90)	4.45 (4.19-4.71)	<0.01
LDL-C, mmol/l	3.01 (2.95-3.06)	3.27 (3.18-3.36)	3.19 (3.12-3.27)	2.90 (2.65-3.15)	<0.01
HDL-C, mmol/l	1.40 (1.38-1.43)	1.39 (1.35-1.44)	1.36 (1.33-1.39)	1.20 (1.12-1.29)	<0.01
TG, mmol/l	1.40 (1.33-1.48)	1.60 (1.48-1.72)	1.62 (1.53-1.71)	1.69 (1.51-1.87)	<0.01
non-HDL-C, mmol/l	3.17 (3.11-3.23)	3.51 (3.41-3.60)	3.45 (3.37-3.53)	3.24 (2.99-3.50)	<0.01
FG, mmol/l	4.92 (4.88-4.96)	5.24 (5.16-5.31)	6.79 (6.58-7.01)	7.24 (6.72-7.76)	<0.01
HbA1C, %	5.82 (5.80-5.84)	6.34 (6.24-6.43)	6.79 (6.66-6.91)	7.35 (6.99-7.71)	<0.01
SBP, mmHg	129.9 (129.0-130.8)	140.5 (139.2-141.9)	148.2 (146.5-149.8)	142.0 (137.7-146.2)	<0.01
DBP, mmHg	80.4 (79.8-81.0)†	83.3 (82.4-84.1)	84.8 (84.0-85.7)†	82.7 (80.3-85.1)	<0.01
Women, n					
% (n)	48.30 (1065)	23.36 (515)	25.66 (566)	2.68 (59)†	
Lipid Profile					
TC, mmol/l	5.09 (5.03-5.14)‡	5.44 (5.36-5.53)	5.20 (5.11-5.28)‡	5.01 (4.75-5.26)‡	<0.01
LDL-C, mmol/l	3.37 (3.31-3.42)‡	3.69 (3.61-3.76)	3.41 (3.34-3.498)†	3.29 (3.07-3.51)‡	<0.01
HDL-C, mmol/l	1.57 (1.54-1.59)‡	1.52 (1.48-1.55)	1.46 (1.43-1.49)	1.45 (1.36-1.53)†	0.01
TG, mmol/l	1.52 (1.46-1.58)†	1.79 (1.71-1.88)	1.97 (1.83-2.11)‡	1.80 (1.57-2.03)†	<0.01
non-HDL-C, mmol/l	3.52 (3.46-3.58)	3.91 (3.83-3.99)	3.74 (3.664-3.832)	3.55 (3.30-3.81)	<0.01
FG, mmol/l	4.98 (4.95-5.01)	5.37 (5.31-5.43)	7.60 (7.38-7.82)‡	7.83 (7.21-8.45)	<0.01
HbA1C, %	5.88 (5.86-5.89)	6.40 (6.32-6.67)	7.25 (7.12-7.38)	7.57 (7.26-7.88)	<0.01
SBP, mmHg	132.7 (131.8-133.6)†	146.7 (145.4-148.1)	144.0 (142.5-145.5)†	141.2 (136.6-145.8)	<0.01
DBP, mmHg	79.8 (79.3-80.3)†	83.3 (82.5-84.0)	82.2 (81.4-82.9)†	82.5 (80.1-84.9)	<0.01

Values are mean and standard error (X ± SE), or percentages % (number).

10-year estimated risk groups were for low (<10%), moderate, (10-20%), high (≥ 20%, or established CVD or diabetes) and very high risk (established CVD plus diabetes) group.

SBP, systolic blood pressure; TC, total cholesterol; LDL-C, low-density lipoprotein cholesterol; HDL-C, high-density lipoprotein cholesterol; Non-HDL-C, sum of LDL-C and VLDL-C calculated as total-C minus HDL-C; TG, triglyceride; FG, fasting glucose, BMI, body mass index.

*Statistical comparison for linear change across 10-year estimated risk low to very high-risk groups, P<0.01

†Statistically significantly different from men, p<0.05; ‡Statistically significantly different from men, p<0.01

Supplement table 3：Chinese Guideline of TC or LDL value of Dyslipidemia patients to start TLC and medication and the target value

Risk stratification	Start TLC	Start treatment	Target value
Low risk：<br>10 yr estimate ＜10％	TC≥6.22mmol/L<br>(240 mg/dl)<br>LDL-C≥4.14mmol/L<br>(160 mg/dl)	TC≥6.99mmol/L<br>(270 mg/dl)<br>LDL-C≥4.92mmol/L<br>(190 mg/dl)	TC ＜6.22mmol/L<br>(240 mg/dl)<br>LDL-C＜4.14mmol/L<br>(160 mg/dl)<br>non-HDL-C<4.92mmol/L<br>(190 mg/dl)
Moderate risk：<br>10 yr estimate 10～20％	TC≥5.18mmol/L<br>(200 mg/dl)<br>LDL-C≥3.37mmol/L<br>(130 mg/dl)	TC≥6.22mmol/L<br>(240 mg/dl)<br>LDL-C≥4.14mmol/L<br>(160 mg/dl)	TC＜5.18mmol/L<br>(200 mg/dl)<br>LDL-C＜3.37mmol/L<br>(130 mg/dl)<br>non-HDL-C<4.14mmol/L<br>(160 mg/dl)
High risk：CHD or CHD equivalent,<br>or 10 yr estimate ≥20%	TC≥4.14mmol/L<br>(160mg/dl)<br>LDL-C≥2.59mmol/L<br>(l00mg/dl)	TC≥4.14mmol/L<br>(160mg/dl)<br>LDL-C≥2.59mmol/L<br>(l00mg/dl)	TC＜4.14mmol/L<br>(160 mg/dl)<br>LDL-C＜2.59mmol/L<br>(100 mg/dl)<br>non-HDL-C<3.37mmol/L<br>(130 mg/dl)
Very high risk：ACS or ischemic<br>cardiovascular diseases with diabetes	TC≥2.59mmol/L<br>(100 mg/dl)<br>LDL-C≥1.81mmol/L<br>(70mg/dl)	TC≥2.59mmol/L<br>(100mg/dl)<br>LDL-C≥1.81mmol/L<br>(70mg/dl)	TC＜2.59mmol/L<br>(100 mg/dl)<br>LDL-C＜1.81mmol/L<br>(70 mg/dl)<br>non-HDL-C<2.59mmol/L<br>(100 mg/dl)

TC, total cholesterol; LDL-C, low-density lipoprotein cholesterol; Non-HDL-C, sum of LDL-C and VLDL-C calculated as total-C minus HDL-C.

Supplement table 4: Statin treatment and control of dyslipidemia in low to very high CVD risk of Chinese subject aged over 65 years stratified by gender

	Low risk (n=1828)		Moderate risk (n=866)		High risk (n=1131)		Very High risk (n=125)	
	no statin	statin	no statin	statin	no statin	statin	no statin	statin
**Men**								
Number	738	25	344	7	522	43	42	24
Statin treatment, % (n)		3.27 (25)		2.00 (7)		7.61% (43)		36.36 (24)
Total TC not at goal	3.93 (30)		38.46 (135)		75.75 (428)		96.96 (64)	
TC not at goal, % (n)	4.06 (30)	0 (0)	38.66 (133)	28.57 (2)*	77.77 (406)	55.81 (24)**	95.23 (40)	100 (24)
Total LDL-C not at goal	7.47 (57)		42.16 (148)		75.57 (427)		81.81 (54)	
LDL-C not at goal, % (n)	7.58 (56)	4.00 (1)	42.44 (146)	28.57 (2)*	77.01 (402)	58.13 (25)**	88.09 (37)	70.83 (17)*
TG>1.7 mmol/L, % (n)	22.22 (164)	24.00 (6)	30.81 (106)	28.57 (2)	31.03 (162)	25.00 (9)	42.85 (18)	50.00 (12)
HDL-C, % (n) <1.2mmol/L	12.19 (90)	4.00 (1)	11.91 (41)	0 (0)	13.60 (71)	18.91 (7)	23.80 (10)	25.00 (6)
Total Non-HDL-C not at goal	3.27 (25)		23.36 (82)		51.32 (290)		69.69 (46)	
Non-HDL not at goal, % (n)	3.38 (25)	0 (0)	23.54 (81)	14.28 (1)*	52.87 (276)	32.55 (14)**	73.80 (31)	62.50 (15)
**Women**								
Number	1038	27	496	19	524	42	36	23
Statin treatment, % (n)		2.53 (27)		3.68 (19)		7.42% (42)		38.98 (23)
Total TC not at goal	11.07 (118)		62.52 (322)		85.86 (486)		98.30 (58)	
TC not at goal, % (n)	11.17 (116)	7.40 (2)	63.70 (316)	31.57 (6)**	86.45 (453)	78.57 (33)*	100 (36)	95.65 (22)
Total LDL-C not at goal	17.74 (189)		65.82 (339)		83.03 (470)		91.52 (54)	
LDL-C not at goal, % (n)	17.91 (186)	11.11 (3)	67.13 (333)	31.57 (6)**	84.92 (445)	59.52 (25)**	97.22 (35)	82.60 (19)*
TG>1.7 mmol/L, % (n)	28.70 (298)	44.44 (12)	40.72 (202)	36.84 (7)	47.32 (248)	43.24 (16)	41.66 (15)	43.47 (10)
HDL-C, % (n) <1.0mmol/L	17.63 (183)	18.51 (5)	18.54 (92)	21.05 (4)	29.18 (153)	17.80 (13)	16.66 (6)	26.08 (6)
Total Non-HDL-C not at goal	6.57 (70)		39.41 (203)		63.42 (359)		83.05 (49)	
Non-HDL not at goal, % (n)	6.66 (68)	7.40 (2)	40.12 (199)	21.05 (4)*	64.69 (339)	47.61 (20)*	86.11 (31)	78.26 (18)

10-year estimated risk groups were for low (<10%), moderate, (10-20%), high (>20%, **established CVD or diabetes)** and very high risk (**established CVD plus diabetes**) group.

*, Statistically significantly different from non-statin treatment group, p<0.05; **,p<0.01;

Supplement Table 5: Detailed Lipid Lowering Medication in Chinese Subjects Aged over 65 Years and Older

% (n)	All participants (N=3950)
Statins	5.32% (210)
Atorvastatin	60.95 (128)
10 mg	**67.97 (97)**
20mg	19.53 (25)
Simvastatin	21.90 (46)
20mg	71.73 (33)
Xuezhikang	10.48 (22)
Others	6.67 (14)
